# F72. NEUROCOGNITION AND ADAPTIVE FUNCTIONING IN THE 22Q11.2 DELETION SYNDROME MODEL OF SCHIZOPHRENIA

**DOI:** 10.1093/schbul/sby017.603

**Published:** 2018-04-01

**Authors:** Fiksinski Ania, Elemi Breetvelt, Jacob Vorstman, Eva Chow, Erin Lee, Lisa Palmer, Erik Boot, Nancy Butcher, Rene Kahn, Anne Bassett

**Affiliations:** 1 UMC Utrecht; 2 University of Toronto; 3 UMC Utrecht, University of Toronto

## Abstract

**Background:**

Identifying factors that influence functional outcome is an important goal in schizophrenia research. These factors, including overall cognitive functioning (IQ) and more specific domains of neurocognitive functioning, may not only aid in identifying those individuals at greatest risk for poor functional outcome but could inform potentially targetable treatment objectives. The 22q11.2 deletion syndrome (22q11DS) is a unique genetic model with high risk (20–25%) for schizophrenia. This study aimed to identify potentially targetable domains of neurocognitive functioning associated with functional outcome in adults with 22q11DS.

**Methods:**

Using data available from a comprehensive battery of 15 neurocognitive tests for 99 adults with 22q11DS (n=43 with schizophrenia) we derived four domains of neurocognition (Verbal memory, Visual memory, Motor functioning, and Executive performance) using a principal component analysis. To investigate the association of these domains with adaptive functioning, we used Vineland Adaptive Behavior Scales (VABS) data available for 84 subjects in a logistic regression model that accounted for the effects of schizophrenia status and overall intellectual level.

**Results:**

The regression model explained 46.8% of the variance in overall functional outcome (p < 0.0001) and 47.7% of the variance on the daily living skills subdomain (p < 0.0001). Executive performance was significantly associated with subsequent functional outcome (p = 0.046); age and schizophrenia were also significant factors. VABS adaptive functioning scale scores were higher in those with better performance on Executive domain tests, no psychotic illness, and older age. The effects of Executive Performance on functioning did not significantly differ between those with and without psychotic illness.

**Discussion:**

The significant relationship between Executive Performance and functional outcome is a novel addition to our understanding of cognitive factors that may contribute to the variability in functional outcome in schizophrenia high-risk groups. The results provide impetus for further studies of Executive Performance as a potential target of early intervention strategies to mitigate risk for schizophrenia and functional deterioration.

